# Supplementary material for: Causal Relationship between Aging and Anorexia Nervosa: A White-Matter-Microstructure-Mediated Mendelian Randomization Analysis
Source: Biomedicines. 2024 Aug 16;12(8):1874. doi: 10.3390/biomedicines12081874 (PMC11351342; doi:10.3390/biomedicines12081874)
Supplement: Supplementary file 1 [file biomedicines-12-01874-s001.zip › Supplementary Figure/Figure S4/Singe SNP plot of MR analysis for TL to ubm-b-1823.pdf]

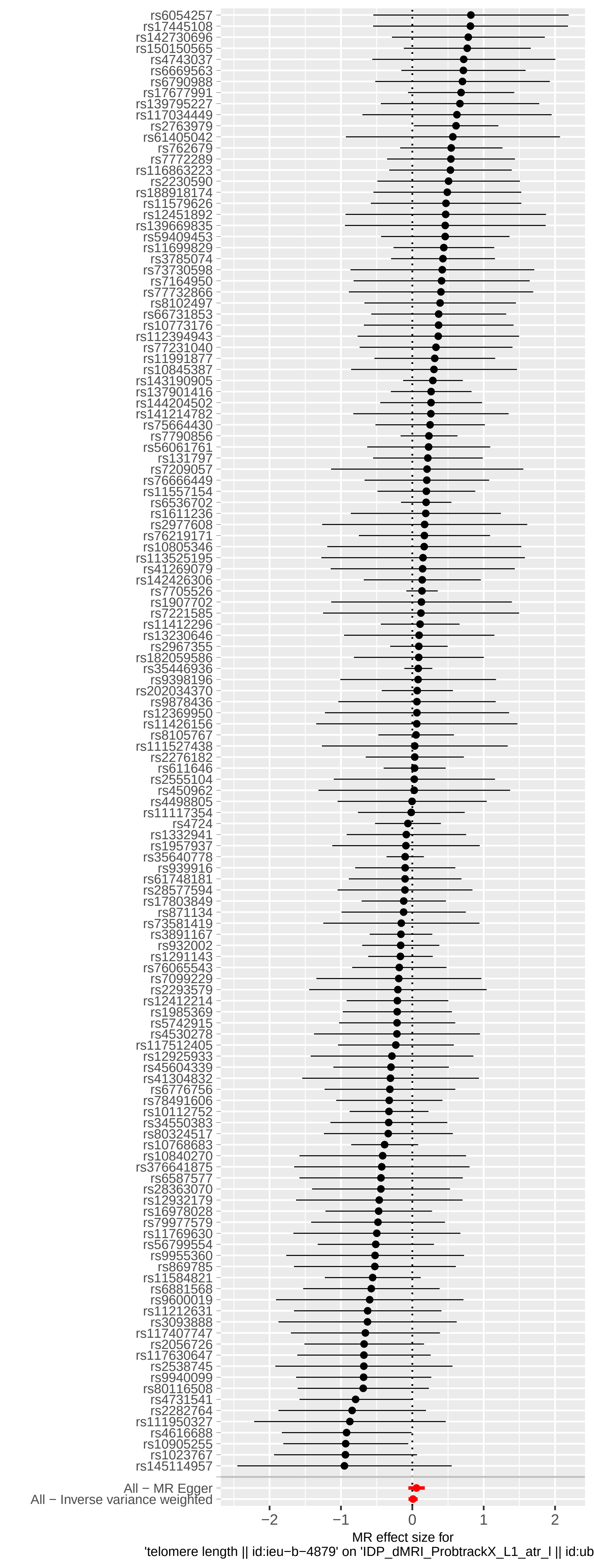
rs6054257
rs17445108
rs142730696
rs150150565
rs4743037
rs6669563
rs6790988
rs17677991
rs139795227
rs117034449
rs2763979
rs61405042
rs762679
rs7772289
rs116863223
rs2230590
rs188918174
rs11579626
rs12451892
rs139669835
rs59409453
rs11699829
rs3785074
rs73730598
rs7164950
rs77732866
rs8102497
rs66731853
rs10773176
rs112394943
rs77231040
rs11991877
rs10845387
rs143190905
rs137901416
rs144204502
rs141214782
rs75664430
rs7790856
rs56061761
rs131797
rs7209057
rs76666449
rs11557154
rs6536702
rs1611236
rs2977608
rs76219171
rs10805346
rs113525195
rs41269079
rs142426306
rs7705526
rs1907702
rs7221585
rs11412296
rs13230646
rs2967355
rs182059586
rs35446936
rs9398196
rs202034370
rs9878436
rs12369950
rs11426156
rs8105767
rs111527438
rs2276182
rs611646
rs2555104
rs450962
rs4498805
rs11117354
rs4724
rs1332941
rs1957937
rs35640778
rs939916
rs61748181
rs28577594
rs17803849
rs871134
rs73581419
rs3891167
rs932002
rs1291143
rs76065543
rs7099229
rs2293579
rs12412214
rs1985369
rs5742915
rs4530278
rs117512405
rs12925933
rs45604339
rs41304832
rs6776756
rs78491606
rs10112752
rs34550383
rs80324517
rs10768683
rs10840270
rs376641875
rs6587577
rs28363070
rs12932179
rs16978028
rs79977579
rs11769630
rs56799554
rs9955360
rs869785
rs11584821
rs6881568
rs9600019
rs11212631
rs3093888
rs117407747
rs2056726
rs117630647
rs2538745
rs9940099
rs80116508
rs4731541
rs2282764
rs111950327
rs4616688
rs10905255
rs1023767
rs145114957
All − MR Egger
All − Inverse variance weighted
−2
−1
0
1
2
MR effect size for
'telomere length || id:ieu−b−4879' on 'IDP_dMRI_ProbtrackX_L1_atr_l || id:ub
